# Differential scanning calorimetry: An invaluable tool for a detailed thermodynamic characterization of macromolecules and their interactions

**DOI:** 10.4103/0975-7406.76463

**Published:** 2011

**Authors:** Michael H. Chiu, Elmar J. Prenner

**Affiliations:** Department of Biological Sciences, University of Calgary, T2N 1N4 Calgary, AB, Canada

**Keywords:** Differential scanning calorimetry, drug, macromolecule, lipid, antimicrobial peptide, drug development, pharmaceutical, drug characterization, nanoparticles

## Abstract

Differential Scanning Calorimetry (DSC) is a highly sensitive technique to study the thermotropic properties of many different biological macromolecules and extracts. Since its early development, DSC has been applied to the pharmaceutical field with excipient studies and DNA drugs. In recent times, more attention has been applied to lipid-based drug delivery systems and drug interactions with biomimetic membranes. Highly reproducible phase transitions have been used to determine values, such as, the type of binding interaction, purity, stability, and release from a drug delivery mechanism. This review focuses on the use of DSC for biochemical and pharmaceutical applications.

Differential Scanning Calorimetry (DSC), is a straight forward, non-perturbing technique, first developed in the early1960s. This method measures the thermodynamic properties of thermally induced transitions and has been applied to a variety of biological macromolecules such as lipids or proteins.[1,2] Examples of these applications have involved conformational states of proteins, DNA binding to protein,[3] biopolymer melting, lipid phase transitions, and lipid-protein interactions.[1,4]

Differential Scanning Calorimetry is primarily used to determine the energetics of phase transitions and conformational changes and allows quantification of their temperature dependence.[5] Technical improvements over time have resulted in high sensitivity instruments, which also make DSC a very relevant tool for investigating the thermodynamic properties of various pharmaceutical products, such as, biopolymers, proteins, peptides, and lipid carriers.[1,4]

Many reviews are available on protein conformation,[4] biopolymers stabilization,[6] thermodynamic properties of lipids,[1] and lipid-protein interactions,[7] however, this article will focus on the application of DSC in the pharmaceutical field, with an emphasis on drug-lipid interactions. Many groups have made relevant contributions and no overview can be fully comprehensive to acknowledge that. Most references in this article are reviews that will provide the reader with sources for a wealth of detailed references.

## History

**Figure 1:**
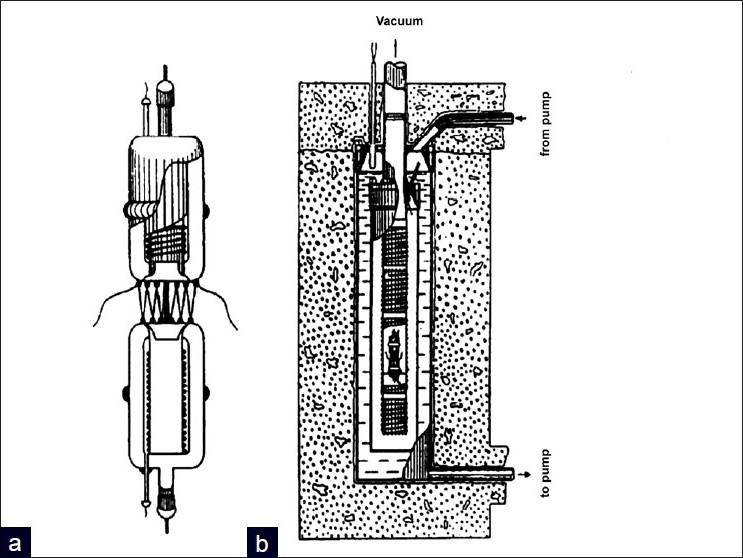
a) Figure of the first DSC used for studying liquids b) Schematic of an adiabatization system using thermal shields, vacuum and water jackets. Reprinted from Thermochimica Acta, Vol. 139, P.L. Privalov V.V. Plotnikov, Three generations of scanning microcalorimeters for liquids, 257-277, 1989, with permission from Elsevier

The evolution of scanning microcalorimeters has progressed rapidly since first described in a publication in 1964 [Figure 1a].[8] Initially designed for measuring temperature-induced heat-release from conformational changes, the instruments were applied to biopolymers and the melting of DNA double helices. The introduction of differential adiabatic scanning microcalorimeters (DASM), in 1963, allowed continuous measurements of heat capacity as a function of a set heating rate.[8] Adiabatic processes are defined as the absence of heat transfer between a system and the environment, and early DSCs used shields, vacuum or water jackets to protect temperature feedback loops to the outside environment [Figure 1b].[8] Moreover, the continuous measurement over a set temperature range was a major advancement allowing for the comprehensive analysis of temperature dependence on thermally induced events.[8] Furthermore, the development of differential heating abilities enabled comparison of the energy difference between a reference and sample cell, which effectively canceled contributions from extraneous factors or solvents.[8]

The next key breakthrough was the miniaturization of the cells, which improved the sensitivity, by drastically reducing temperature gradients that occurred in larger samples. Moreover, this design change also allowed the elimination of stirring mechanisms.[9] By replacing cylindrical cells with capillary tubes that had a very high surface-to-volume ratio, the effects of viscosity and gradient heating were minimized. Furthermore, the capillaries could withstand higher internal pressure than other cells of the same thickness. The increased pressure resistance increased the operational temperature and made DSC a very versatile tool, with a large temperature range.[8,9]

Subsequently, non-adiabatic differential scanning microcalorimeters were designed, as they were simpler to manufacture and were more applicable in the industry. The use of cells that were removed for washing and required adiabatization after loading resulted in baseline instability and irreproducibility. Moroever, altering the heat capacity measurements from mass to volume minimized the large error associated with loading, and increased the accuracy and reproducibility of the machine (more details in the next section).[8,9] The cells were fitted with sensors to determine the volume, rather than relying on user measurements.[9]

Two main systems are used to control cell temperatures. The first is a power compensation unit, which independently controls and monitors the temperature of the reference and sample cells. Constant energy is provided to both cells, hence, the temperature increases at a steady rate.[10] However, a thermally induced transition that requires heat results in a temperature lag in the sample cell compared to the reference. The extra heat required to maintain the same temperature between two cells is used to calculate the excess heat capacity.[10] Independent controls utilize two heating / cooling units (one for each cell) to maintain the temperature. The second system is referred to as a heat-flux or heat leak principle, where both cells are connected via a low resistance heating flow-path (usually a metal disk). The recorded difference in voltage of the temperature-measuring device is proportional to the temperature difference that is used in the heat capacity calculation.[10]

There are multiple different designs of scanning calorimeters based on the applications and samples tested, however, they all share three main characteristics.[3] The first is the fact that calorimeters must be able to measure temperature changes, keep constant heating or cooling rates, and take accurate temperature measurements.[2,3] Second, the instruments must accurately measure the differential heat flow between the sample and reference cell, which results in better baseline stability and reduced noise.[3,9] Finally, the cell contents are measured usually in volume (older instruments may still use mass), which is essential for reproducible and accurate values.[3]

The various modern calorimeters also retain twin cells and a differential heating mode, where cells are heated or cooled quasi-adiabatically at a constant rate.[5] Current calorimeters have become exceptionally more accurate with advancements in sample size, baseline stability, and sensitivity.[3,5] The temperature range of operation has also been increased using high pressure to scan to about 100°C and super cooling to measure below 0°C.[3] Moreover, many different DSC models are available based on their application. Examples include Hyper DSC, which allow very high scanning rates such as 400 – 500°C/min and modulated DSC, which can separate heat flows from reversible and non-reversible events. Nano DSC can operate with very small quantities of sample per trial, approximately 130 µL or 100 µg, while maintaining the accuracy of larger volume calorimeters.[11,12] In recent times, fully automated cleaning and loading devices have been incorporated in many DSC models, which enable computer-controlled sample addition, cell cleaning, and sample degassing. Such instruments can test 50 samples a day with increased accuracy and minimal systematic errors.[11,13]

## Theory

Differential Scanning Calorimetry is used to measure the specific heat capacity of thermally induced events as a function of temperature.[5] The apparent specific heat (c_2_) of a solution is calculated by the following equation:

(1) $$c_{2}\;=\;c_{1}\;+\;1/w_{2}\left(c-c_{1}\right)$$

where c is the specific heat of the solution, c_1_ is the specific heat of the solvent, and w_2_ is the weight fraction of the solute.[4] DSC measures the excess apparent specific heat (c_ex_), which is the value (c-c_1_) in equation 1. Expanding the definition of c_ex_ (c-c_1_), the measured heat capacity of the buffer (c_1_) can be written as:

(2) $$C_{b}\;=\;m_{b}\times {C_{b}}^{{}^{\circ}}$$

where m_b_ and C_b_° are the mass and the specific heat capacity of the buffer, respectively. Equally, the heat capacity of the sample solution (c) can be expressed as:

(3) $$C_{s}\;=\;m_{s}\times {C_{s}}^{{}^{\circ}}$$

with ‘s’ denoting the sample. By subtracting these two values the c_ex_ can be determined.[2] The value (m_b_-m_s_) can be replaced by the partial specific volume, removing mass from the equation, as new calorimeters use the more precise volume over mass measurements.[2]

The differential heat flow from the calorimeter is temperaturedependent and is referred to as a thermoanalytical curve. As the scan rate is constant, the time integral of the measured differential heat flow provides the energy of the sample.[3] As the c_ex_ is usually quite small (about 0.7% for a 1% aqueous protein solution), using equal volumes of solution and proper shielding from external effects is of paramount importance.[4] The excess specific heat is plotted against temperature, revealing the respective transitions. Integration of c_ex_ over the temperature range results in specific calorimetric enthalpy ∆h_cal_.[10] However, traditionally, problems arise when performing integrations.[2,4] For example, the course of the baseline is not necessarily obvious during a phase transition and may change after the transition, thus, artificial baselines and sophisticated software tools are necessary.

## Experimental Procedures

Sample preparation differs depending on the type of sample to be analyzed although in most cases the compound of interest is studied in buffered aqueous solutions. Sharp peaks such as the first order gel-to-liquid crystalline phase transition (L_α_) seen for high purity phosphatidylcholines (PC)[4] require very low scan rates of around 0.1 K min^-1^ or less, so as to avoid the broadening commonly seen with faster scan rates. Slower scan rates are also beneficial as they enhance the resolution, thus enabling the resolution of closely spaced DSC peaks that may arise from single phospholipid phase transitions. However, slow scanning rates result in decreased signals and more sensitive calorimeters are required.[4]

Most modern DSC instruments have two cells one as a sample and one as a reference, but some calorimeters have three samples cells that can be scanned against the same reference.[10] As the volume is used to determine the c_ex,_ sample and reference solutions are normally degassed prior to being loaded into the cell. This is important, to avoid the formation of bubbles that will affect the accuracy of the volume and add spikes and experimental noise to the thermograms. However, a disadvantage is that the small capillary cells will make the cleaning more difficult, which may also result in bubble formation.[8]

State-of-the-art instruments allow setting a variety of experimental parameters such as the post scan temperature, the number of scans, their range, scan rate, and feedback strength. As discussed earlier, slower scan rates provide higher resolution. Furthermore, a high feedback will give optimal sensitivity to the reaction, but may increase the noise levels in the experiments. Finally, it is important to reach equilibrium before the thermotropic data are analyzed. To ensure that this has been reached, sufficient scans are recorded until two scans are superimposed.

Once the parameters have been chosen for an experiment, the temperature is scanned at the set rate in the heating or cooling mode. Initially the temperatures in both cells increase linearly to the same extent, resulting in a zero baseline.[1,10] However, once the sample undergoes a phase transition a temperature difference is observed. During endothermic events the recorder will move upward, indicating that energy input is required and in an exothermic event a downward deflection is seen as less energy being required from the DSC, to maintain the temperature. The size of the deflection is dependent on the heating or cooling rate, and following the thermal event, the signal returns to baseline or a new baseline can be detected if there has been a change in the specific heat.[1]

## Analysis

Differential Scanning Calorimetry analysis is performed on equilibrium data.[4,14,15] Depending on the system investigated, different means of analysis and different models have been devised. Most interpretations are based on the van’t Hoff equation:

(4) $$\left(d\ln\;K\;/\;dT\right)\;=\;\Delta H_{vH}/\;{\mathrm{RT}}^{2}$$

where K is the equilibrium constant of the process, T is the absolute temperature, and ∆H_vH_ is the van’t Hoff enthalpy.[4,14] This equation is only applicable to two state processes, without significant intermediate populations, during the transition.[4] This model is normally applied as most systems have an initial state, some intermediate state during the transition, and a final state. There are even differences in the two state models based on whether there is a change in specific heat after the transition, as observed for the denaturation of the T4 lysozyme.[4] More complex models are also used for multi-state changes such as gradual unfolding and the presence of different intermediate states that make data analysis more complicated. In such cases a different equation, incorporating the entire transition, is utilized, where each step has its own set of parameters, such as, van’t Hoff Enthalpy and T_1/2_. For a comprehensive review on the different models refer to.[2,4,14]

**Figure 2:**
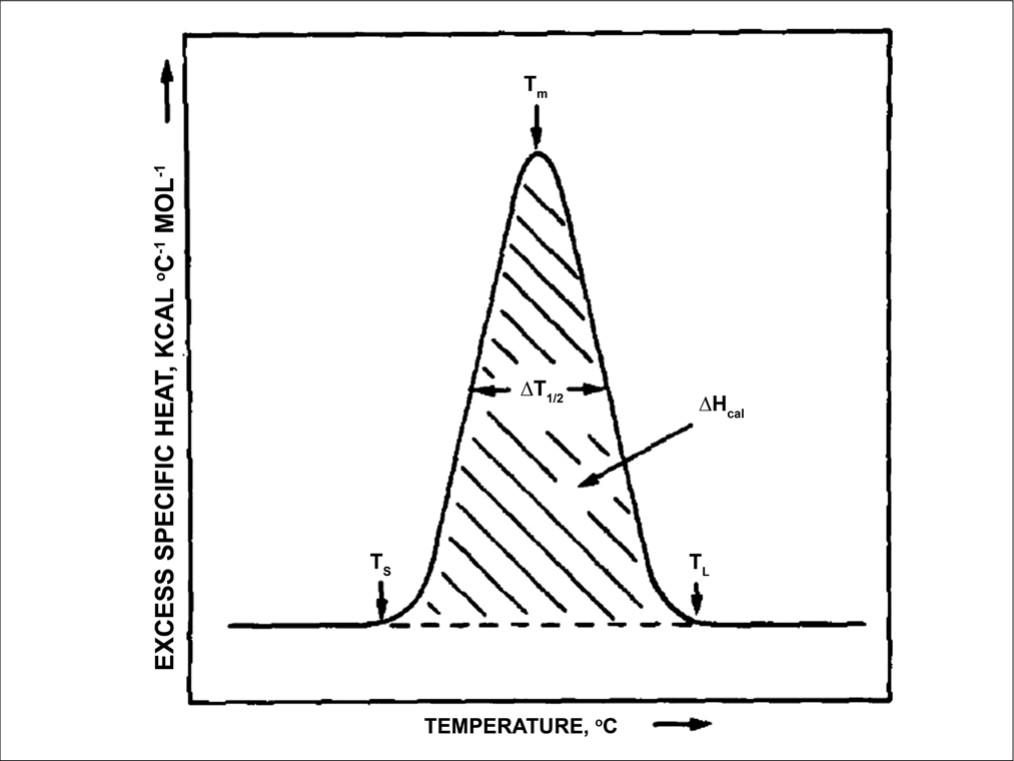
Enthalpy, T_1/2_ and T_m_ shown on a DSC endotherm. Reprinted from Chemistry and Physics of Lipids, Vol. 30, R.N. McElhaney, The use of differential scannning calorimetry and differential thermal analysis in studies of model and biological membranes, 229-259, 1982, with permission from Elsevier

The enthalpy of the endothermic or exothermic event is determined by the integration of the area under the DSC peak, which is often reported in kcal / mol [Figure 2].[1,10,14,16] Initially this was performed by means of a planimeter or even by cutting and weighing the paper traces, to determine the values to use in the van’t Hoff equation.[1,4] Today, various iterative processes in the modern software are used, with different equations, based on the type of process (two state, irreversible, etc.). Moreover, the instruments are calibrated with known standards and a buffer blank is subtracted to provide accurate enthalpy values.

The maximum height of the transition (also maximum heat capacity) occurs at the phase transition.[1,16] In the case of lipids the peak of a symmetrical curve represents the temperature at which the gel-to-liquid-crystalline state is half complete [Figure 2]. However, many biological extracts and pure phospholipid thermograms are asymmetrical and the T_m_ is not longer the midpoint of phase transition,[1] and in this case the width of the distribution is considered (see a little further in the text).

The shape of the thermally induced event is described by the width of the transition at half height of the peak (T_1/2_), whereby, the peak is defined by the difference between the lower (T_S_) and upper boundaries (T_L_) of the phase transition [Figure 2]. Values can range from 0.1°C for pure phospholipids to over 15°C for biological membranes.[1,16] T_1/2_ is a valuable tool to gauge purity, protein-lipid interactions, as well as lipid-lipid interactions, and provides information about the cooperativity of the phase transition.[10]

Cooperativity of a pure lipid transition is related to the shape and sharpness of the peak and is described by a cooperative unit (CU), the number of lipids involved in the transition.[10] Furthermore, CU can be calculated by the ratio of ∆H_vH_/∆H_cal_, where ∆H_cal_ is the enthalpy of the transition (cal / mol) and ∆H _vH_ is the van’t Hoff enthalpy.[4] The van’t Hoff enthalpy can be determined using an approximate relationship relation: H_vh_ ~ format 4RT_m^2^/T_1/2 (equation 4).[1] For a purely cooperative first order transition, cooperativity would reach nearly infinity, whereas, a non-cooperative process will reach zero[1] Highly purified synthetic phospholipids can yield almost fully cooperative transitions, but as even small impurities can have a significant impact, the cooperativity value should be interpreted with caution.[1,16]

Besides the three main values immediately apparent from the DSC trace, other important thermotropic parameters can also be calculated. As free energy (G) is zero at the phase transition T_m_, the enthalpy can be calculated using the equation

(5)^[10]^ $$\Delta S\;=\;H_{Cal}\;/\;T_{m}$$

Where H_cal_ is the enthalpy that corresponds to the area under the transition peak.

Moreover, the partition function for a macromolecule system can be found by a double integration of the apparent heat capacity.[4] Fractional occupancy of different states has also been calculated, based on DSC thermograms, assuming that only two distinct states exist.[10] This is performed using the equilibrium constant:

(6)^[10]^ $$K=\;\left[B\right]\;/\;\left[A\right]\;=\;f\left(1-f\right)$$

where K represents the equilibrium constant, A and B are the respective states, and f is the fractional occupancy.[10] As K can be determined via:

(7)^[10]^ $$\Delta G^{{}^{\circ}}\;=\;-\mathrm{RT}\ln K\;=\;\Delta H^{{}^{\circ}}\;-\;T\Delta S^{{}^{\circ}}$$

the absolute heat capacity difference between the unfolded and folded states can be used to show solvent accessible polar and apolar surfaces between the states.[2] Deconvolution analysis of the heat capacity function can yield the number of states that will be populated during the denaturation of the protein, which allows a more detailed analysis of this process.[17,18] For a full review on protein analysis using DSC refer to review.[2]

## Applications

Considering the ability to measure enthalpy changes and phase transitions, there are multiple applications for such a versatile tool. There are good reviews on its application to proteins,[3] protein for pharmaceutical interest,[19] protein mutations,[20] protein-ligand interactions,[11,21,22] protein folding,[23–26] nucleotides,[4] other macromoleculesdont,[6,27] lipids,[28,29] drug-lipid interactions,[30] and protein-lipid interactions.[31] This review will start with a brief overview of other pharmaceutical applications and will focus on lipid-drug interactions such as antimicrobial peptides. A good review on drug development using DSC is presented in[32–34] and for drug development uses for DSC.[33]

## Proteins

As pharmaceutical products can come in the form of proteins (e.g., enzymes), their thermodynamic properties are important, and one of the earliest DSC applications was to study thermally induced, cooperative conformational changes of small proteins.[6,35,36] However, small molecules do not yield good data unless they aggregate, showing intermolecular cooperation. The application of DSC to protein denaturations was described by Freire and Biltonen,[37,38] who reported that thermal transition was synonymous with the protein partition function, suggesting that the thermogram can be used to identify the states in denaturation.[37,38] Thus, protein thermodynamics, during unfolding, is measured as an enthalpy change, as a function of temperature, to determine the partition coefficient.[14] For a full review of the thermodynamic calculations for different types of denaturation see.[4,14,35,36,39]

Differential Scanning Calorimetry-based analysis of the thermal denaturation of proteins provides an insight into the unfolding process and forces involved in conformation stability.[4,40] For comprehensive reviews on protein denaturation refer to[3,4,41] and for protein folding.[14,42] During protein denaturation there are different thermodynamic states, with many microscopic states. This process is highly cooperative with disruption of many forces and bonds, including hydrogen bonds, hydrophobic interactions, and many non-covalent interactions.[41] DSC allows for the direct study of thermal stability, over a very large concentration range, in the absence of light, thus photosensitive proteins such as bovine lens crystallins can be analyzed.[41]

Conversely protein folding can also be studied, investigating thermotropic changes in different environments. The energetics and heat capacity, ∆C_p,_ of the protein, refolding into different conformations such as α-helix or β-barrel structures[25] is used for this purpose. Such analysis has been performed on the α-helical membrane protein, bacteriorhodopsin, which yields a high transition temperature and low unfolding enthalpy.[25] For a review on the energetic states of protein conformations, refer to.[25] Furthermore, the enthalpy of relaxation (∆H ^*^) can be investigated by using DSC for the characterization of the structural relaxation of a protein.[43]

Heat capacity for thermally induced protein denaturation has revealed thermodynamic information about the different states,[9,35] as it depends on three major factors. The first relates to the primary structure of the protein and contributes from stretching and bending to the rotating of internal bonds.[6,35,36] The second factor is based on non-covalent interactions from the secondary and tertiary structures. Finally contributions from the hydration affect the heat capacity. The primary structure provides the most significant contribution, followed by hydration, and less impact from the non-covalent secondary and tertiary interactions.[6]

Such denaturation processes can be categorized into either two-state denaturations or multi-state denaturations. The former can be further broken into multiple different groups, such as, those with self-dissociation, ligand dissociation, and large permanent specific heat changes.[4] Multi-state denaturation has been observed for many proteins, including histones H1 and H5.[4,36] Different trends in the T_m_, T_1/2_, and enthalpy are observed, and hence, allow the classification of a given protein. Once the denaturation process has been established, the stabilizing factors and conformations can be more easily assessed.

Accompanying protein denaturation is the study of protein stability, which is of great importance in understanding its role and in its screening, for improved stability of proteins, for pharmaceutical applications.[37] DSC can be used to study two types of protein stabilities, thermodynamic stability or kinetic stability.[37] Most calorimetric protein studies involve the thermodynamic stability, relating to the equilibrium between the native folded and the unfolded or partially unfolded states.[37] The focus on thermodynamic stability is due to the ease of studying small proteins and the availability of software and algorithms that can be easily applied.[37]

Kinetic stability relates to the Gibbs energy between the folded and unfolded states, reached with progressive scans. The amount of time required to adopt the proper state or lose the adopted conformation is essential in pharmaceutical applications, for drug shelf life and potency. Furthermore, the ability to adopt the active state under non-ideal intracellular and extracellular environments may vary and different environments can be investigated using DSC.[37] Low kinetic stability drugs have been improved through mutations, and reassessed using calorimetry, as a quick comparison with the original can be performed. Moreover protein-ligand stability has also been studied to screen for undesirable effects such as aggregation or proteolysis.[11,12,37,44] A current review has discussed the role of DSC in the kinetic stabilization of proteins.[11,12,37]

The solid state of proteins has also been studied using DSC, as the chemical and physical degradation is significantly reduced. The solid state provides the ability for improved drug delivery without the need of an excipient.[45] However, DSC is better suited for proteins dissolved in a solution. This has been directly applied to investigate the propensity of liquid protein therapeutics, to aggregate during storage.[46] DSC allows for a quick scanning procedure to detect the presence of aggregates without the need for extended stability trials.

However, problems arise when studying protein denaturations, as the majority of transitions are deemed calorimetrically irreversible, as upon denaturing, a subsequent scan will show no transition or a significantly reduced one.[37,47] Most protein denaturation analyses are performed assuming equilibrium thermodynamics, hence, suitable analysis is only available for kinetic stability.[37] Further problems with the conformational analysis of proteins relate to dilute sample solutions of the protein. The high background heat capacity of the system may overshadow signals from dilute samples and require very sensitive and precise calorimeters.[2,9,35] Good reviews on applications of proteins and potential problems associated with calorimetry are presented in.[11,12,47]

**Figure 3:**
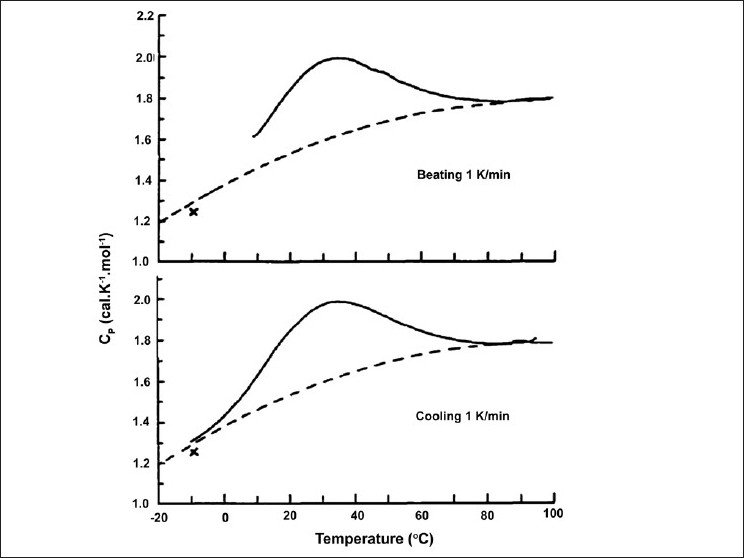
Heating and Cooling Scans of an alpha helix forming peptide. Reprinted from Methods in Enzymology, Vol. 323, G.P. Privalov P.L. Privalov, Problems and prospects in microcalorimetry of biological macromolecules, 31-62, 2000, with permission from Elsevier

Applications of DSC for proteins are not limited to structural changes, as shown in a few examples. The polymerization steps of tobacco mosaic virus coat protein include thermally induced reversible conformational changes, which can be investigated by both heating and cooling scans [Figure 3].[4,12,35] DSC has also been used with proteins of the plant photosynthetic system, to study the effects of temperature on the heat inactivation process of photosystem 2.[6] Moreover, molecular recognition studies have been performed by DSC as ligand or drug association alter intermolecular interactions, which result in changes of T_m_, enthalpy and free energy associated with such interactions.[21,48] Such protein ligand studies have been reported for glucose transporter GLUT-1 and ATP as well as bovine serum albumin and anilinonaphtalene sulfonate (ANS)[48]

Protein-lipid interactions such as the interaction of various apoproteins with different lipid mixtures have been investigated showing preferential binding to specific matrices.[49,50] Studies on the lipid interaction of cytochrome C oxidase showed that one oxidase molecule perturbed over 70 lipid molecules corresponding to the lipids surrounding the protein.[51] For a review on membrane proteins and DSC refer to.[51] Protein effects on surfactant lipid systems have also been studied with SP-B and SP-C.[52] Furthermore, the binding stability has shown that most DNA-binding proteins are typically unstable without DNA.[3] This is used to determine the conformation of the protein upon binding DNA.

## DNA Drugs

Base stacking enthalpies and helix-coil enthalpies have been used to determine conformations of DNA.[4] Generally it has been found that an increase in enthalpy of 8 - 10 kcal (mol / base pair)^-1^ is observed for a helix coil transition.[4] These enthalpies have even been used to predict quaternary and quinternary structures of DNA in liver nuclei.[53] The investigation of the heat capacity of DNA and RNA identified water clusters on the nucleic acid matrix.[6] A change in hydration can be used to explain the exposure of polar or apolar groups revealing the possible drug-binding sites.[3] For a review on DNA thermogram analysis refer to.[54]

**Figure 4:**
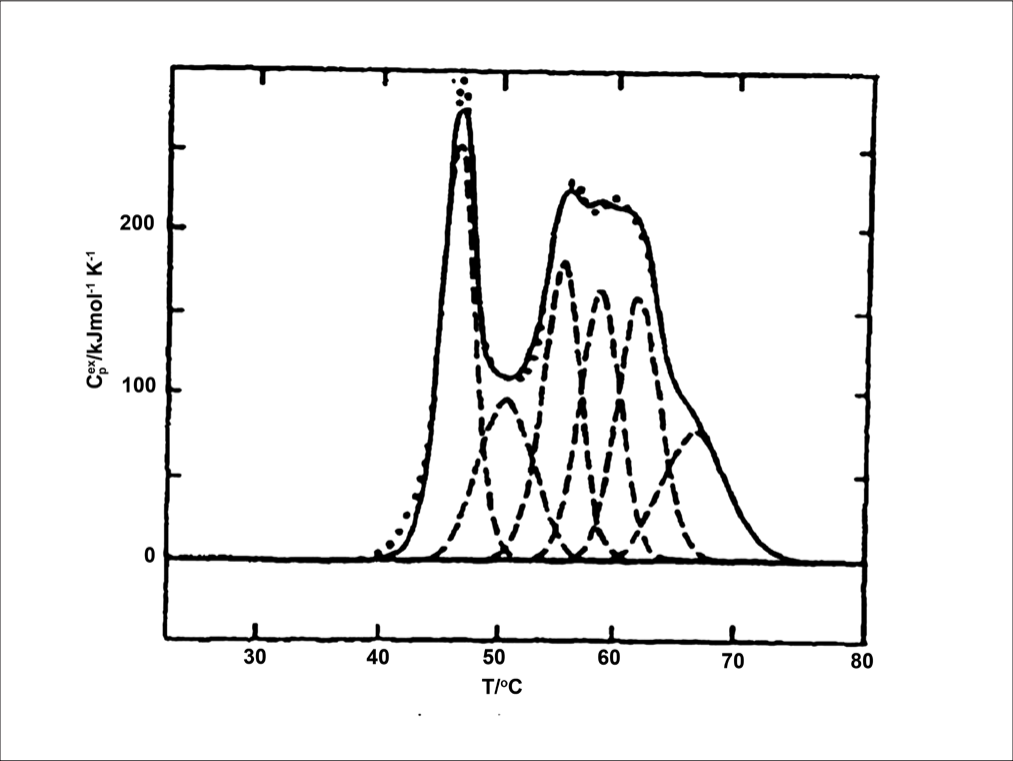
Deconvolution of a thermogram with (.....) representing experimental data and (-----) representing the deconvoluted data for excess heat capacity of myosin rod in 0.5M KCl, 0.2 M Phosphate pH 7.0. Reprinted from Thermochimica Acta, Vol. 193, G. Castronuovo, Proteins in aqueous solutions. Calorimetric studies and thermodynamic characterization, 363-390, 1991, with permission from Elsevier

Many of the DNA melting curves are typically quite broad and contain overlapping regions,[55] because DSC only measures the overall enthalpy changes and cannot distinguish between enthalpies from different thermodynamic events. Statistical deconvolution has been applied to many of these thermograms, by essentially ‘desmearing’ low resolution overlapping transitions by fitting them to individual peaks that contribute to the enthalpic endotherm.[41] Deconvolution has been utilized to provide a direct means of obtaining a partition function and properties of intermediate states [Figure 4].[18,54] First described by Freire and Biltonen, deconvolution can be used to establish the partition function of the thermal unfolding event by using a mathematical algorithm.[18,38,54] Once the partition function is determined, properties such as cooperative melting and information about more complex structures such as oligomeric hairpins, can be analyzed.[54] Typical deconvolution of DNA melting profiles yields biphasic and triphasic transitions and allows for a thermodynamical description of the transitions for each complex, by indicating the favorable enthalpic contribution due to base stacking and the effects of environment, such as, pH and ionic strength.[55]

Thermal stability of nucleic acids and their melting behavior in various duplex or triplex conformations has been studied using DSC.[55] Denaturation of triplex structures has shown that initially the third strand is removed followed by the unfolding of the duplex.[17,55] Furthermore, a melting analysis of the different oliognucleotides revealed forces involved in the structural stability as well as the effect of ions, pH, and temperature.[6] Ligand-DNA interactions similar to protein-ligand interactions have been used to test the pharmaceutical development potential of anti tumor drugs, by assessing their binding to DNA.[4] For a review of the calorimetric binding of anti-tumor drugs to DNA, see.[4]

Differential scanning calorimetry has also been applied to analyze *in vitro* interactions of antitumor drugs, with human epithelial cell nuclei that exhibit a characteristic melting profile, with four structural transitions.[56] A loss of the fourth transitional peak upon drug treatment is correlated with the inhibition of cell division induced by different DNA strand breakers and alkylating drugs. The primary mode of action of many antitumor drugs is DNA damage and cleavage and the observed changes to the four transitions provides an insight into the mechanism of breakage.[56] Hence, DSC is a quick screening tool to observe the effect of intercalating drugs on a nucleosome structure. Changes in the supercoiled loops can also be used to study DNA strand breakage and to assess the effect of intercalating drugs on base pair stability. This has been observed for belomycin and streptonigrin, which destabilize the supercoiled DNA to a relaxed form, characterized by a drop in enthalpy and the T_m_ of the fourth transition.[56] Moreover, different mechanisms of DNA interaction can be elucidated as alkylating drugs produce a kinetic intermediate peak, and intercalating drugs reduce the melting temperatures of transition II, but increase the T_m_ of transition IV.[56]

Intercalating drugs from the anthracycline group of antibiotics such as ethidium bromide and actinomycin D showed a characteristic shift of the seven DNA melting peaks observed in a plasmid, to higher temperatures.[57] The magnitude of the shifts depended on the strength and concentration of the drug. Furthermore, the binding sites could be determined from the peaks that diminish with increasing concentration.[57] Examples include the binding of danomycin to the 5’CG-3”-rich region of DNA sequences.[57] This insight can help to distinguish between minor or major groove binders and to identify other specific binding sequences, which aid in rational drug design.

DNA-drug interactions have been studied for many non-steroid anti-inflammatory drugs (NSAIDs).[58] Variations in the calorimetric data such as enthalpies and temperatures for the unfolding of DNA provide information about the type of drug interaction. For example a primarily electrostatic interaction results in a decrease in enthalpy with increased drug addition.[58] The overall stability of the DNA is affected by many compounds that impact the observed scans. A stabilizing effect (e.g., seen for urea) will shift the calorimetric peak to higher temperatures.[58,59] Furthermore, the presence of more Guanine-Cytosine base pairs increases the enthalpy due to additional hydrogen bonds that stabilize the double helix. Addition of NSAID drugs such as naproxen and ketoprofen lower the T_m_, which indicates a reduction in the energy required for denaturations, suggesting a destabilizing drug interference between base-pair interactions.[58]

Moreover, the effects of novel methods such as virus-induced gene silencing can also be investigated by DSC. The treatment of many genetic disorders is envisioned via the delivery of plasmid DNA, which has been studied *in vitro* and *in vivo*.[60] Typical DNA transfection techniques suffer from cellular toxicity and the safety of such retroviral delivery systems is not well-established. pH-sensitive liposomes have been utilized as potential plasmid delivery mechanisms.[60] Plasmid pPTCK-6A was encapsulated in a DOPE / Cholesterol and an antigen, resulting in immunoliposomes. The gene was shown to be successfully delivered into the cell by monitoring the reporter gene.[60] DSC can be used to monitor the interaction of the plasmid with the liposome and to ensure that aggregation does not exist.

## Lipids

Phospholipids are one of the most studied lipids by DSC.[61] One of their major advantages is that pure synthetic phospholipids undergo transitions at well-defined temperatures based on their structure.[10,61] Hence, the transitions are easily reproducible and trends can be established within systematically altered lipids (e.g., progressively increasing chain length). Pure lipids are analyzed as aqueous dispersions, formed from a lipid film, by mechanical agitation, such as vortexing. They contain multilamellar vesicles (MLV), which are closed multi sheaths comprised of concentric bilayers that are separated by aqueous spaces.[1,62,63] MLVs are the predominate form used to investigate lipids, as they provide the clearest resolution of phase transitions with accurate enthalpy values.

Different vesicle preparations alter the observed thermograms, as single unilamellar vesicles (SUVs) produce a lower resolution peak than MLVs. Sonicated disaturated PC thermograms reveal less enthalpic transitions with a greater T_1/2_ and no notable pre-transition. The increase in peak width is likely from a reduced enthalpic component rather than a decrease in CU. However, a decrease in cooperativity can be attributed to the smaller radius of SUVs over MLVs, resulting in a less ordered orientation, which increases the free motion of the hydrocarbon chains.[1] The affect of the radius coincides with other calorimetric studies indicating that the thermogram is dependent on the size of the vesicle.[10] When DPPC SUVs are studied in the cooling mode their main T_m_ decreases to 37°C (MLV 41°C) accompanied by a lower enthalpy and a substantially larger T_1/2_.[10,64] However, complications arise when the SUVs are studied in the heating mode, as they tend to fuse by forming large unilamellar vesicles (LUVs). The thermograms of LUVs are nearly identical to those of MLVs, although with slightly broader endotherms attributed to the size inhomogeneity.[10,64]

**Figure 5:**
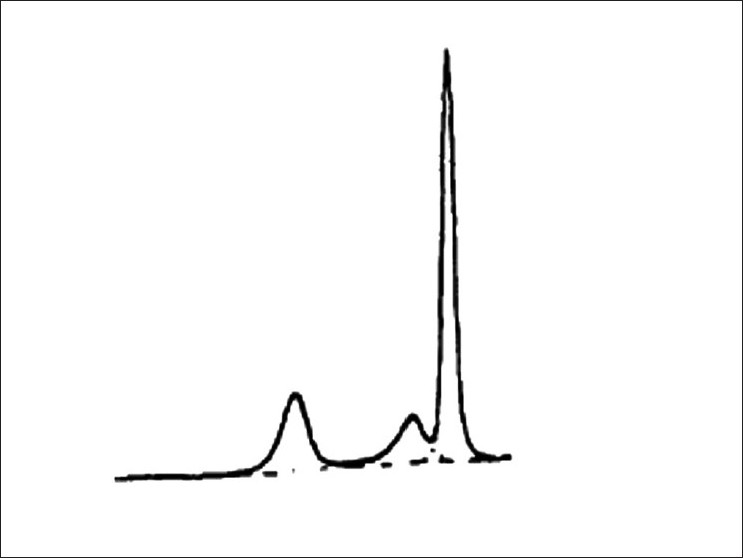
DSC Heating endotherm for DPPC MLV. All three transitions can be seen. (DPPC was equilibrated at 5°C for 2 days prior). Reprinted from Biochemistry 24, M. J. Ruocco, D. J. Siminovitch, and R. G. Griffin, Comparative Study of the Gel Phases of Ether- and Ester-Linked Phosphatidylcholine, 2406-241, 1985. With permission from American Chemical Society

Multilamellar vesicle thermograms produce reversible and highly cooperative transitions Phospholipids exhibit three main groups of phase transitions, however, they are not always detectable [Figure 5].[10] The first is the most observed and best characterized gel-to-liquid crystalline transition, L_α_, which occurs at the T_m_. This transition is quite rapid and is the conversion from a gel to liquid crystalline state.[10] The second transition is only seen for some phospholipids and usually occurs below the T_m_. It is much slower and exhibits much less enthalpy when compared with the L_α_. This so called pre-transition is from a gel to a rippled gel phase and is sensitive to impurities and has been used to gauge vesicle preparation.[10] A review on using DSC to evaluate liposome preparation is available.[10] The last transition is not very well characterized and usually occurs below the operational range of most conventional DSCs. This subgel transition is very slow and does not reveal a lot of thermodynamic information.[10] Each of these transitions is characterized by its own temperature (T_m_, T_p_, T_s_, respectively), their own enthalpy (∆H _m_, ∆H _p_, ∆Hs respectively), and their own half width T_1/2_.[10] The gel-to-liquid crystalline lipid phase transition is the most well-understood, however, the DSC data has also suggested that the pre-melting and pre-freezing phenomena can provide information about the liquid-liquid phase separation and boundary defects in the solid state.[1]

The main transition is where the lipid membrane changes from a relatively ordered crystalline-like gel state to a disordered fluid-like state.[1,61] This transition is due to the cooperative melting of the hydrocarbon chains, which retains the lamellar structure. It includes a conformational change of the hydrocarbon chains from all trans in the rigid gel state to a disordered state that allows gauge conformations.[1] Accompanying these changes in hydrocarbon orientation, are a lateral expansions due to increased mobility, and a concomitant decrease in the bilayer thickness.[1] Moreover, the increasing chain length and saturation, results in higher enthalpy values for the L_α_ transition.[6] Hence the phase transition enthalpy of lipids depends on the structure of the lipid, especially with the position of unsaturated bonds and length of the fatty acid chain.[1,6] Moreover, shifts in T_m_, enthalpy, and increased T_1/2_ values are good indicators of sample purity and liposomes size distribution.[10] Many of the thermodynamic properties of synthetic and biologically derived lipid phase transitions are available through an online database LIPIDAT http://www.lipidat.chemistry.ohio-state.edu[65]

## Phosphatidylcholine

Phosphatidylcholines (PCs) are among the most common components of mammalian membranes[65] and have mainly structural roles. A comprehensive review of the phase transitions of PC is available.[10,65] The most important thermodynamic event is the gel-to-liquid crystalline transition, which is a two-step, first-order endothermic process.[1,65] Fully saturated phosphatidylcholines (PCs) with identical fatty acid tails are among the most common lipids studied by DSC and exhibit sharp and symmetric chain-melting transition. The more commonly studied lipids (DMPC)(di-14:0) and DPPC (di-16 : 0) exhibit peaks at 24°C and 41°C [Figure 5].[10,65–67] Depending on the temperature range, scan rate, and fatty acid length (> 14 carbons), a pre-transition peak (T_p_) that is typically of lower enthalpy and broader endothermic transition than L_α_ can be seen.[1,10,65] The temperature interval between the pre-transition and the main transition peaks decreases with increasing fatty acid chain length and both coincide at about 22 carbons.[1,65,68] However, the values reported for pre-transition show more disparity than the L_α_ data, due to a larger dependence on the scan rate and often the values are higher than the equilibrated data. The affects of the scan rate on T_p_ are stronger for PCs with more than 16 carbons.[1,10,65] Furthermore, even minor additions or impurities diminish or abolish the pre-transitional peak. The associated heat (∆H_cal_) is between 1.0 and 1.8 kcal / mol, and this transition is highly cooperative, involving several hundred lipids, independent of the chain length.[1,10,65]

Figure 5 illustrates all three transitions in a DSC scan of DPPC. From left to right one can find sub-transition (T_s_ = 21°C), pre-transition (T_p_ = 36°C), and the main transition (T_m_ = 41.3°C).[10,68] These values are dependent on the scan rate, as lower scan rates have resulted in lower T_p_ temperatures. PCs found in biological membranes, have both saturated and unsaturated fatty acid tails and exhibit considerably lower T_m_ values compared to disaturated PCs.[1,65] For 1-palmitoyl-2-oleoyl phosphatidylcholine (POPC), the T_m_ values have been reported to be between -5°C and 3°C, with an enthalpy (∆H _cal_) of approximately 8 - 8.1 kcal.mol. Although there is a significant difference between the reported T_m_ of POPC and the disaturated DPPC (~41°C), the ∆H_cal_ values for both are similar.[1,10,65] These PC bilayers have been used as model eukaryotic systems.[69] Typically unsaturated PCs are not studied using DSC, as the main transition falls below the operating range of most instruments, however, a review on mixed acyl chain PCs is available.[70]

The impact of double bonds on the lipid phase behavior depend on their location and type.[10,65] Trans double bonds tend to have fewer effects on lipid packing than cis double bonds.[10,65,71] A systematic DSC-based screen of double bond position in an unsaturated fatty acid shows a characteristic U-shape when the T_m_ versus the double bond position is plotted. The minimum T_m_ is found when the double bond is in the center of the fatty acid chain.[1,10] This trend also holds true for the ∆H_cal_ with higher enthalpy found when the double bond is located at the beginning or the end of the fatty acid. Cis double bonds tend to decrease in order, which results in increased entropy due to an increase in free volume and the rotational degree of freedom, which is not seen for trans double bonds. This results in larger decreases of T_m_ for lipids containing cis double bonds.[10,65] The decreasing T_m_ directly affects entropy as follows:

$$T_{m}\;=\;\Delta H^{{}^{\circ}}\;/\;\Delta S^{{}^{\circ}}.$$

[10]

Differential scanning calorimetry has also been used to investigate the effect of different salts on the thermotropic behavior of the PCs. Monovalent cations such as Na^+^ or K^+^ did not show much affect, even at high concentrations (1 M), on the properties of the pre- or main transitions.[1,65,72] However, the divalent Mg^2+^ and Ca^2+^ substantially changed the lipid phase behavior of the lipids.[73] 1M Mg^2+^ increased the melting temperature of the pre-transition, the main transition, and the enthalpy, whereas, low concentrations of Ca^2+^ (1 mM) have been shown to decrease the enthalpy of the pre-transition. The effects of Ca^2+^ are considerably stronger compared to Mg^2+^, as concentration above 10 mM induce a substantial increase of T_p_ and a moderate increase of T_m_.[1,65] At large concentrations (250 mM Ca^2+^) the pre-transition and main transition peaks merge together. Salt affects are more commonly seen for negatively charged phospholipids, as zwitterionic PCs tend to be less sensitive to cations.[1,65,72] The combination of the lipid head group structure as well as pH, salt concentration, and ionization states affect the thermodynamic properties.[10]

## Other Lipid Classes

The chemical structure of the polar head group affects the L_α_ transition via hydrogen bonding capabilities and electrostatic interactions.[1,6,10] DSC results for the polar and zwitterionic phosphatidylethanolamine (PE) vary with pH, due to different protonation states, however, most studies are done at neutral pH values and show consistent trends. Disaturated PEs have a higher T_m_ than the corresponding PCs due to the hydrogen bonding capabilities of the PE headgroup that adds stability.[1,74] Furthermore, the smaller headgroup of PE allows for closer interactions of the lipid molecules resulting in a more stabilized gel state. The T_m_ values increase with increasing chain length, similar to PC, and similar ∆H_cal_ values were reported for both disaturated PCs and PEs.[1,68] However, contrary to PC the disaturated PEs do not show any pre-transitions and an asymmetric main transition is evident. Differences in enthalpy have been observed for di-unsaturated lipids, whereby, the PE values are approximately half of what is seen for the corresponding PCs.[1,74] The cooperativity of the main transition is also reduced as disaturated PEs have CU values that are only about half of those for the equivalent PCs. Contrary to PC it has been found that due to the extra hydrogen bonding capabilities from NH_3_^+^ and PO_4_^-^ between separate bilayers, a tight interaction is formed, reducing the hydration levels,[74] in contrast to many other lipids. DSC has been used to analyze the hydration energetics of different PC and PE bilayers and the impact on the non-lamellar properties.[74]

The pH of the solution will affect the T_m_ of the transition depending on the protonation / deprotonation state of the amino group. Deprotonation at low pH reduces the hydrogen bonding capabilities, and thus, decreases the T_m_ of DPPE from 63°C to 41°C.[74] Moreover, pH values below 8 increase the propensity for PEs undergoing a sharp transition from a lamellar to a hexagonal (H_11_) phase, within or above the L_α_ temperature.[74] The enthalpy of the non-lamellar phase is not easily detected by DSC and thus pH and temperature range needs to be considered when studying PEs.[1]

Phosphatidylethanolamine can form either a lamellar or hexaganol phase depending on the type of acyl chains.[74–76] Short diacyl chains typically less than 12 carbons form lamellar phases, whereas, unsaturated systems yield hexagonal conformations, with the lamellar to H_II_ phase transition dependent on the number of hydrocarbons and frequency and position of the unsaturation.[74,77,78] PE lipids have a cylindrical shape, indicating equal size from the tails and headgroup, typically from short, fully saturated, hydrocarbon tails. A cone-shaped structure caused by a larger lipid tail region and smaller headgroup preferentially adopts a hexagonal phase.[74,75,79] The lamellar to hexagonal transition (T_H_) is found to occur at a minimum temperature when the unsaturation is closest to the middle of the acyl chain, as seen with T_m_ and PC.[80] The lowest T_H_ corresponded to an unsaturation at position 9, similar to the reported findings for the L_α_ transition for PCs and PEs.[80] Moreover, the non-lamellar properties of PE are being harnessed as a possible drug delivery mechanism, such that lipid-based nanoparticles incorporate PE hexagonal phase transition for drug release.[77] DSC has been found to show transition to hexagonal phases with higher sensitivity than ^31^P NMR or X-ray scattering, making it an ideal choice for many of the different drug studies.[78] An extensive review on the calorimetric behavior of different PE species is presented in[77] with the kinetics of PE transitions described in.[81,82] Furthermore, many different calorimetric studies have been compiled into the LIPIDAT database.[77]

Phosphatidylglycerol (PG) is a major component of mitochondrial and chloroplast inner membranes as well as a pulmonary surfactant, but not a main structural component of mammalian membranes.[83,84] However, PG along with PE is one of the major lipids in bacterial membranes.[83,84] Thermograms of negatively charged PGs have generally been found to correlate well with PCs, as corresponding disaturated species sharing similar T_m_, ∆H_cal_, and entropy values.[1] The pre-transitional peak coincided with the PC studies, with disaturated PGs having a pre-transition with similar thermotropic properties and an absence of pre-transition, with di-unsaturated species.

However, ion concentration and divalent Ca^2+^ and Mg^2+^ induce the formation of metastable complexes with PGs that are not seen with PCs.[85] PG has been found to exhibit a different melting regime with aqueous dispersions of DMPG at pHs higher than the pK_α_ and at high lipid concentrations of 70 – 300 mM, revealing a very broad transition over an interval of about 10°C.[85] There appear to be at least two different phases existing, suggesting that DMPG forms a new phase at higher concentrations.[85] Using other biophysical techniques such as optical microscopy and X-ray scattering this phase has been identified at L_p_ (lamellar with pores), existing 3°C above the T_m_, prior to becoming a fluid phase past 30°C.[85]

In addition to the main phases the stable subgel and the liquid crystalline lamellar phases, L_C_ and L_α,_ there are also metastable gel phases known as L_β’_ and P_β’_ under physiological conditions.[86] Low temperature incubation (4°C) of aqueous DMPG dispersions cause the lipids in the gel phase to transform into a highly metastable ordered solid quasi-crystalline bilayer, particularly for shorter chain lengths.[87] Freeze-fracture morphology has shown that two equal populations of a flat multilamellar sheet and a cylindrical shape occur when the phase transitions are monitored by DSC.[84] Upon cooling below the T_m_ the multilamellar aggregates dissociate forming unilamellar vesicles, which fuse to lamellar stacks upon low temperature storage forming a cylindrical shape.[84] Upon reheating, the main transition is considerably broader due to heterogeneous lipid conformations, and it occurs at a much higher temperature (40.3°C).[84]

Due to polarity and charge of the head group, the pH values and ionic strength become major factors governing the T_m_ of the PG main transition. Low ionic strengths are characterized by a large gel-fluid transition approximately from 18 – 35°C, which produces an optically transparent solution due to rearrangements in lipid packing.[83,88] This results in the transition usually being broken up into different calorimetric peaks called the T_m_^on^ and T_m_^off^, where structural changes occur between.[88] This usually correlates with a sharp decrease in turbidity at T_m_^on^ and an increase at T_m_^off^. The melting process is only fully completed at Tm^off^.[83,88,89] However, the exact structural characteristics of these transitions are still being determined. There is a hypothesis about a three-dimensional bilayer network as a possible structure.[88,89] Low pHs have induced T_m_ increases of 20°C for DPPG, attributed to a minimization of repulsive forces between the negatively charged headgroups. Anionic DMPG vesicles have been investigated with different Na^+^ concentrations, showing ionic-strength-dependent properties.[90] High salt concentrations result in a sharp shape indicating high cooperativity due to the shielding effect of the Na^+^ cation on a negative phosphate group.[90,91] On the contrary, significantly broader transitions were observed in distilled water, due to the absence of shielding.[90,91]

Cardiolipin is a major component of mitochondrial membranes and regulates many different membrane bound enzymes.[92,93] Furthermore, it is present in the bacterial membranes as one of the anionic components.[93] For reviews on the thermotropic characteristics of CL and salt effects refer to.[92,94] CL still retains similar properties to other lipids with an increase in T _m_, as there is an increase in acyl chains.[94] The T_m_ and enthalpy increases with a chain length similar to PG for CL, with the transition temperature being higher for PG.[92] Tetramyristoyl CL has two major endothermic transitions with similar enthalpy, however, the lower temperature transition is less cooperative and shows a cooling hysteresis.[93] CL has a propensity to form HII phases in the presence of high concentrations of salts or a decrease in pH, however, this is dependent on the amount of unsaturation and chain length.[94,95] Salts can be used to convert CL from a lamellar state to an inverted hexagonal phase.[95] CL typically converts to an inverted hexagonal phase at low pH values, when the phosphate group is protonated.[93] Tetraoleoyl cardiolipid showed this shift at NaCl concentrations of 3.5 M or higher.[95] Similar to PG, CL is sensitive to divalent cations, especially Ca^2+^, for which the pre-transition and main transition temperatures are raised.[94]

Calorimetric studies with cholesterol have typically been studied with a lipid mixture check.[72,96] Cholesterol dramatically influences the phase transition by broadening the endotherm, with high concentrations eliminating the L_α_ transition.[72,96] Lower concentrations (<10%) typically induce minimal phase separation. Added cholesterol increases the area of the gel monolayer due to the increasing disorder of the gel bilayer, while ordering the liquid crystalline state.[97] This behavior of the cholesterol facilitates the lamellar formation for many different lipid species including PG, PC, PE, and PS. There is a wealth of calorimetric studies on various cholesterol-lipid mixtures; this section will outline some finding from different mixtures, providing references to more comprehensive reviews.

Two phases in PC and cholesterol mixtures are clearly present at 10 – 25% cholesterol, revealing a liquid ordered cholesterol phase and a liquid disordered phase for the PC.[97–104] This has also been observed with SM.[98,105–109] Mixtures of cholesterol and PG bilayers show complete abolishment of the PG transition at 50% cholesterol for the dimyrstoyl species (DMPG).[99] However, longer acyl chains such as dipalmitoyl persist longer, with remnants of the transition still observable at above 50% cholesterol.[99]

Differential scanning calorimetry of cholesterol has been applied to concepts such as the lipid-raft in cellular membranes and the existence of phase-separated fluid domains in cholesterol-lipid mixtures.[110–114] Cholesterol is one of the key lipids in eukaryotic cells, with essential roles in metabolism, hormone production, and formation of several vitamins.[115] Aside from the possible lipid rafts the role of cholesterol on ordering adjacent lipids has been studied with a recent review presented in.[113,115] Additionally mixtures of PC, SM, and cholesterol have been used to form raft micro-domains, as different concentrations of components result in different phase formations, which can act as potential targeting sites of pharmaceutical products.[96,110,111,114,116–118]

**Figure 6:**
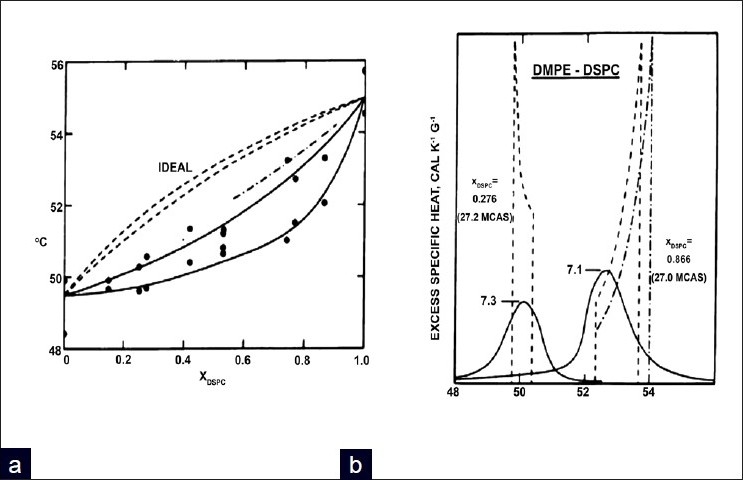
a) A phase diagram showing the deviation from Ideal Mixing b) DSC thermogram of a DMPE DSPC mixture. Reprinted by permission from Proc. Natl. Acad. Sci. 73, S. Mabrey and J.M. Sturtevant (1976) Investigation of Phase Transitions of Lipids and Lipid Mixtures by High Sensitivity Differential Scanning Calorimetry 3862-3866 with permission from Proceedings of the National Academy of Science United States[191]

A good review on calorimetry of lipid mixtures is presented in.[61] Such studies include the thermotropic analyses of the mixture of PC and PG of varying chain lengths, and the hydrate states examined have been at different pH.[83,119,120] Data from these studies have been used to formulate phase diagrams providing information about the mixing behavior of the different systems.[119,121] At neutral pH the phase boundaries are close together with a narrower coexistence between the two compared to pH 2.[119,120] Many comprehensive calorimetric reviews on different lipid mixtures are available from diacylglycerol mixtures with phospholipids,[122] phosphatidylserine, and cholesterol,[123,124] PC and PG,[125] and PE : PG mixtures, with differing chain length and pH.[126] Based on prior studies, mixing lipids with similar thermodynamic properties results in traces that retain similarities to the pure components, however, with an increased asymmetry and T_1/2_.[10] Moreover, the amount of similarity retained in pure components depends on the composition of the two components and the interaction between the polar and non-polar portions.[1,10] This has primarily been done by constructing phase diagrams [Figure 6a]. Phase diagrams use the onset and completion temperatures of phase transitions, the T_1/2_ and enthalpy for different lipid mixtures, and reveal the affect of different compositions on T_m_.[1,10] The comparison to theoretical curves is used to evaluate the phospholipid mixtures.[1] In cases where thermodynamic characters of the lipid components are quite different, the thermogram becomes complex and highly dependent on the concentration, as phase separation and demixing can occur [Figure 6b].[10]

Biomimetic liposome systems have long been used as simplified model membranes for many membrane-drug or membrane toxicity investigations.[28,29,127] A recent review correlates the toxicity of various compounds, such as, xenobiotics, detergents, and peptides, with established toxicity assays showing a good correlation between the two.[127] Analysis of multi-lipid membranes has been used to study biomembranes of eukaryotic cells, revealing domains and organization of lipids, for potential roles in signaling or recruitment.[128,129] Studies of domain components and phase behaviors allows for potential targeting sites for potential pharmaceuticals.[128]

Differential scanning calorimetry has also been used to determine the lateral heterogeneity of membranes as preferential lipid-lipid interactions result in a clustering of lipids.[98] Such data provides an insight into the fluid phase of the membrane, as lateral organization and lipid targets may provide information for potential drug targets.[98,114,130] Clustering of lipid components can be revealed as pure lipid domains demixed in lipid mixtures. Furthermore broadening of the endotherms suggests mixing between the two components.[130] For reviews on lipid domains and calorimetry of different lipid mixtures see,[98,105,131] and in particular.[129]

**Figure 7:**
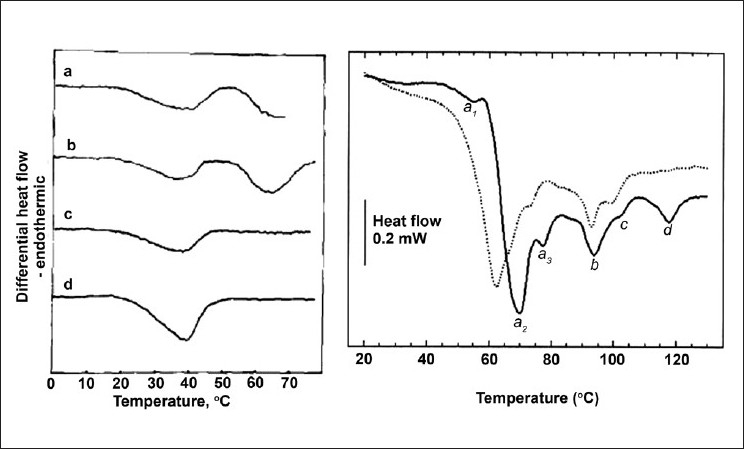
DSC scans of a) A. laidlawii[192] a) whole cell, b) isolated membrane, c) protein denatured, d) aqueous MLV of membrane lipids. b) DSC of whole cell E Coli dashed trace is for E Coli ribosomes. Reprinted from Chemistry and Physics of Lipids, Vol. 30, R.N. McElhaney, The use of differential scannning calorimetry and differential thermal analysis in studies of model and biological membranes, 229-259, 1982, with permission from Elsevier

Calorimetric lipid analysis has also been applied to lipid components in biological membranes, such as aggregates of macrodomains in mammalian blood platelets, in order to evaluate the stability of the platelets during freeze drying, for therapeutic storage.[117] The first successful DSC studies were done on the prokaryote *Acholeplasma laidlawii*,[4] *Halobacterium Halobium*, and unsaturated fatty acid auxotrophs of *Escherichia coli* [Figure 7]. Analysis of the thermotropic data have shown that 90% of the membrane extracts undergo a cooperative transition, even with the multiple lipid species, and membrane protein DSC analysis of *E. coli* membranes showed the absence of a visible gel phase.[98,105,132] For reviews on the heterogeneity of biological membranes and the different mimetic lipid mixtures refer to.[133,134]

## Drug Purity

Physical constants and purity profiles of drugs have been determined by using differential scanning calorimetry.[14,135] The latter can be assessed by the melting behavior observed in the recorded thermograms. Peak integration, according to the van’t Hoff relationship, and T_m_ values are used for batch-to-batch consistency and to test for impurities that will change the melting profile.[135] Although it is difficult to quantitatively measure the percentage or type of impurities, DSC provides a quick and reliable means of establishing batch variability and is a qualitative screen for contamination.[14,135,136] Nevertheless multiple techniques will be necessary to allow proper quantitative analysis.[135,136]

The main application of DSC to purity relies on the notion that impurities reduce the melting temperature of the drug.[137] The melting temperature is a strong indication of drug purity and DSC not only allows for a quick screening of the T_m_, but the resolution of the peak (T_1/2_) will relate to populations of drug that may be in a different conformation or interacting with an excipient resulting in a shoulder region.[14] The amount of impurities is derived from van’t Hoffs’s law for diluted solutions:

$$X\;=\;\left(-\Delta T\;\times \;\Delta H_{f}\right)\;/\;\mathrm{RT}.^{2}$$

with X equaling the mol fraction of impurity, ∆T representative of the melting point depression, T. equal to the melting point of the pure substance, R, the gas constant, and ∆H_f_, the enthalpy of the pure material.[137] Most results have been highly complementary to chromatographic data.[137] Nonetheless, DSC in purity analysis has become increasingly popular, due to the low quantity of sample required (1 – 2 mg) and the relatively quick analysis time.[137]

Another aspect of purity is drug polymorphism, which is related to the different crystalline states.[19,33,138] As pharmaceutical processing results in multiple polymorphs, the bioavailability of the key state of the drug as well as the potential health risks of different states require scrutiny in testing.[33] The polymorphic transitions can be measured using DSC and phase diagrams can be constructed, respectively.[33,121,138] Polymorphs typically exhibit similar properties in the gaseous and liquid states, however, show differences depending on the solid state. The most commonly analyzed states are the amorphous state, crystalline state, and glassy state.[19,138] Amorphous relates to a non-ordered system, whereas, glassy state refers to an amorphous solid that undergoes a glass transition, forming a rubber like appearance.[33] The glass transition (T_g_) is the transition that occurs in amorphous materials, as the heat capacity undergoes a quasi-discontinuous change to a higher value. Another analyzed transition is exothermic crystallization, which occurs as the amorphous solid turns crystalline or semi-crystalline, usually lies in between the glass transition and the T_m._[16] For reviews on distinguishing between amorphous and crystalline states of a drug by DSC, refer to.[43,139]

Amorphous pharmaceutical solids are typically less stable than their crystalline counterparts and the addition of excipients have a tendency to exist as an amorphous solid.[43] Typical pharmaceutical preparative techniques such as lyophilization, milling or wet granulation lead to amorphous conformations.[19,33,139] Hence, DSC has been used to study the thermodynamic differences between the amorphous form and crystalline form as well as to identify a coexistence between both.[43] Crystallization is often exothermic, whereas, amorphous compounds do not recrystallize and the enthalpy recorded can be analyzed quantitatively, to determine the drug state.[14,32,43] The enthalpy of the peak can be used to determine the purity of the peak. As an advantage of DSC over capillary melting point approaches, separate melting transition or polymorphs and recrystallization events can also be observed, which provide information about sample fusion or impurities.[32] In theory, a completely pure crystalline sample should yield an infinitely narrow transition, whereas, increased broadening is associated with impurities.[136] Using van’t Hoff’s law of melting point depression, a straight line is seen when temperature is plotted against the inverse of the molten fraction of a sample.[136] For a full review on van’t Hoff’s law applied to DSC purity profiles, see van Dooren and Muller, 1984.[136] Subsequent to linearization, deviations from a profile expected for a pure compound can also provide information on sample stability. Moreover, other aspects such as changes in sample size or curvature due to the formation of precipitates will affect the validity of the analysis. Furthermore van’t Hoff’s analysis of impurities requires that its contents do not change over time, hence, evaporation or decomposition would affect the results.[136,140] Therefore, different scanning rates are usually compared, to observe the effects of evaporation or decomposition, especially around the melting region.[136] Nevertheless, impurities have been determined to be particularly accurate in 98% of the pure samples of various organic chemicals such as organophosphates, urea, amides, esters or halogenated compounds.[140]

Many pharmaceutical products can be present in different conformations, with distinct chiral structures, which alter their desired effect.[48,141] Racemic compounds usually exhibit different thermal events, therefore, it is possible to detect as low as 1.5% of an isomer in a mixture of almost pure ephedrine hydrochloride.[141] Although difficulties may arise from overlapping thermal events within the same temperature range, deconvolution with non-linear regression has been successful to distinguish isomers.[141] Although there are other methods of determining chiral purity, DSC requires a minimal amount of material, as 1 – 5 mg is adequate for most applications.[141] High sensitivity, reliability, and the relative speed of the assays provide a quick purity screen for different drug batches. This is very valuable for pharmaceutical applications, as isomers possess differential absorption, and altered potency and metabolism or pharmacological behavior.[141]

Moreover, the determination of water and hydrate content is important as most drugs are hygroscopic and the primary solvent for crystallization is water.[32] During the crystallization process of many pharmaceutical compounds, solvents are incorporated into the crystal lattice affecting properties such as solubility, stability, and pharmokinetics.[142] Water content is a critical parameter in drug development as the water activity may vary with different hydrates existing in the same drug product (hydrate polymorphs).[33] Usually determination of the water content is done by thermogravimetric analysis (TGA), Karl Fischer titrimetry (KFT) or evolved gas analysis.[143] However, DSC has been applied, to determine the water stoichometry in different drug hydrates, under the assumption that the enthalpy of dehydration (∆H_d_) is equivalent to the enthalpy of vaporization (∆H_v_) of water.[142] Results correlated well with values from KFT, with the additional benefit of information on the potential location of water binding based on hydration enthalpies. Similar to chirality studies, the technique is limited to overlapping hydration peaks, however, used in conjunction with other techniques it provides a quick and reliable screening method of hydrate content.[142]

## Drug Stability

Product stability is essential and is usually described by the equilibrium constant (K) or the free energy (∆G°).[48] These values can be determined indirectly from the measured enthalpy through thermodynamic correlations such as the van’t Hoff equation,[48] allowing the use of DSC to screen the stability of potential drugs or drug delivery systems. Progressive scans can be used to analyze stability and to assess the denaturation temperature, as gradually changing compounds will yield a different profile. Comprehensive reviews on drug stability, particularly liquid particles are presented in.[42,144,145]

Pharmaceuticals applications of proteins depend on a properly folded state.[48] As a denatured protein has a higher heat capacity than its native form (∆C_p_), an increase in this parameter can be used to determine the extent of denaturation, with progressive cycling, over an extended period of time.[48] Moreover, the melting temperature can also be used as an indicator of thermostability as a higher T_m_ represents a more stable protein that is less susceptible to denaturation.[48]

Drying of proteins for pharmaceutical applications can impact their conformation, and hence, reduce the potency of the drug.[146,147] DSC has been used to gauge different drying techniques for potential pharmaceutical applications. Techniques such as spray drying, lyophilization, super critical fluid technology, and many others have been proven challenged to maintain protein stability under high temperature, freezing, and dehydration.[19,146–149] A more in-depth analysis of these problems is presented in a review,[146] showing how DSC can be used to evaluate potential methods for pharmaceutical protein preparation. DSC also presents an advantage, as a high throughput screening means establishing protein changes quickly and easily, based on different mutations or preparations.[42,144,145]

Lyophilization of liposomes lowers the potential hydrolysis of phospholipids and physical degradation of the vesicles extending the life of the drug carrying molecules.[19,149,150] However, such processes are not without faults, as physical changes may occur, resulting in the damage of the liposomes, releasing the encapsulated agent.[149,150] Lyophilization of liposomes is explained in detail in.[150] Furthermore drug-liposome stability is presented in,[16] with the kinetics of liposome phase transition in.[151,152]

One particular relevant problem for the pharmaceutical industry is drug-excipient interaction.[153] The latter is an inactive substance used to carry the active compound or to minimize drug degradation upon delivery. Drugs and excipients were incubated at a set temperature for a period of time, followed by an increase to a higher temperature, and subsequent isothermal incubation. Analysis of the thermograms will illustrate any changes to the compound, such as, degradation or interactions between the excipient and drug at higher temperatures.[42,153] Furthermore, the sample environment could be easily controlled in the instruments, allowing incubation at high humidity or temperature, to simulate long-term exposure.[153] In addition to the simulated storage of the drug products, choices of different salts and drying methods have been investigated using DSC.[14] The effects of the coating on different drugs and delivery systems such as nanoparticles (mentioned in the next section) have resulted in characteristic shifts and decreases in enthalpy or the T_m._[14] Stability under a wide range of conditions has to be studied, as batch-to-batch variation can result in different polymorphs, as observed with different photochemical stabilities.[33] Problems may arise when using DSC to screen for excipient compatibilities, as it is required to use high temperatures at set heat rates. Hence, inconsistencies between reactions at ambient temperatures and pressurized cells can occur.[34]

The properties of vitamin B6 in different excipients, such as mannitol, were used to gauge changes to the properties of the drug.[154] In conjunction with other highly sensitive thermal techniques, such as Micro-thermal analysis (µTA), the changes in thermotropic properties could be used to select the best suited excipient.[154] The study of cyclodextrins as an excipient has been of great interest in the pharmaceutical field as the torus-shaped, cyclic structure allows for encapsulation of drug molecules inside a less hydrophilic cavity, compared to the aqueous solvent[155] Three different drugs trimethoprim, sulfadiazine, and sulfamethoxazole, with natural cyclodextrins (α,β,γ) were studied in both the aqueous and solid states, showing lower stability of the drugs in the amorphous state and solubilizing properties depending on the carrier size of the cyclodextrin.[155] DSC-derived excipient compatibility is usually compared to spectroscopic results obtained by UV or IR or to chromatographic HPLC analysis.[156]

Differential scanning calorimetry has been very effective in determining the physiochemical properties of different pharmaceutical products, thus facilitating design of new drugs or improving modifications of the existing compounds.[154] With the abilities to test both drug and excipient for purity, stability or pharmacological properties, DSC is becoming increasingly popular in the pharmaceutical industry.[154]

## Nanoparticles

Thermal analysis can also be used to analyze the incorporation of drugs into nanoparticles via examining enthalpy change.[15,157–159] Liposomes have been used to penetrate skin for drug delivery and localized drug delivery.[160] DSC is one of the primary tools used for the characterization of the matrix state, with polymorphism and drug incorporation in lipid dispersions.[161] Nanoparticles tend to have a decreased melting temperature compared to bulk material that is not in the nanometer size.[161] Lipid polymorphism is commonly found in lipid nanoparticle dispersions with various components affecting molecular packing, which is reflected in the different melting points and enthalpies.[161] Furthermore, the smaller radius prevents optimal lipid packing of the lipid acyl chains, thus lowering the energy required for the phase transitions. Broadened profiles are usually attributed to the addition of multiple different lipid components, as well as size differences.[161] For a review on liposome drug delivery refer to[162] and for nanoparticle drug interactions.[163]

Analysis of drug loading efficiencies is quite complicated, as the drug typically interacts with the lipids inducing a shift in the phase transition temperature.[161] Moreover, the enthalpy of the transition may also be reduced as a population of lipids is interacting with the drug solubilized in the matrix.[161] This can easily be used to identify if the drug is miscible in the melted state of the liposome. Most studies presume changes to the lipid thermogram and a negative shift of the matrix lipid T_m_ to be a sign of drug incorporation. However, in some cases it has been reported that decreases in enthalpy can be attributed to lipid dissolution or aggregation of drug molecules within the nanoparticles.[161]

Improved efficacy of different drugs has been studied using nanoparticle delivery systems.[164] A potent cancer fighting drug, Paclitaxel, has difficulties in administration, due to poor solubility in water and with excipients. Nanoparticles composed of biodegradable polymers with poly(lactic-co-glycolic acid) have been used to encapsulate the drug within the nanoparticles, using emulsifiers such as cholesterol and phospholipids.[164] DSC allowed for comparison of the thermodynamic properties, as the T_m_ of Paclitaxel and the nanoparticle carriers were analyzed, to screen for undesirable changes to the drug.[164] DSC was also used to record the transition of non steroid anti-inflammatory drugs (NSAIDs) from a crystalline to an amorphous state upon encapsulation in polyethylene glycol (PEG), a solid drug carrier, accompanied by a decrease in endothermic transition over time and progressive scanning.[165]

Similar studies were performed with solid lipid nanoparticles (SLN) prepared from oil-water microemulsions to encapsulate the drug diazepam.[166] Solid lipid nanoparticles are emerging as a potential application in drug delivery, due to their low toxicity and their ability to maximize drug incorporation for secondary and tertiary drug targeting.[157,158] Thermograms of crystalline diazepam and the drug loaded SLN particle, showed that the melting peak for the drug was not observed in the loaded nanoparticles, indicating an amorphous solid in the SLN.[166] Moreover, the solid state particles can exist in polymorphs, pseudopolymorphs, and even amorphous solids.[167,168] DSC melting profiles are essential for identifying the state of the drug, which can significantly influence bioavailability, stability, and water content.[157,169] This is essential for determining the proper state, for the active pharmaceutical ingredient.[169]

Relating to drug stability, the lipids in SLNs are considered excipients, with different lipids and surfactants studied and presented in a recent review.[170] Thermotropic analysis of the SLN particles indicated that the chemical stability of the lipid is not affected during formation with a low level of degradation (2–5%) for the majority of lipids and a maximum of 10% reached after 24 months.[170] However, other lipid excipients such as lecithin have shown strong decomposition, minimizing its potential in SLN.[170] Structural properties and thermodynamic characteristics of nanocrystallization have been studied in detail in.[139]

In addition, DSC was used to analyze stability and drug dissolution from nanostructure lipid carriers (NLC) composed of a solid lipid matrix with a liquid lipid nanocompartment core.[159] Drug release from three-dimensional polymer hydrogel systems is a growing field in biomedical drug delivery.[171] Site-specific targeting and release increases the bioavailability. Thus, it is important for pharmaceutical testing, to understand the interaction between nanoparticle carriers and drugs as well as nanoparticles and biological membranes. Different nanoparticle polymers and various cross-linkers have been used to modulate temperature-dependent drug release *in vitro* and some have been shown to obstruct drug diffusion and incorporation.[171]

The application of dendrimers for drug delivery to the lungs was probed by studying their interactions with DPPC liposomes, as the latter is the main component of a lung surfactant.[52] Changes to the lipid phase transition are used to determine properties such as incorporation of the dendrimer into the bilayer as well as the strength of interaction, based on the overall structure and hydrophobicity of the dendrimer.[172]

The manufacturing of plastics and rubber relies on plasticizers, to enhance the flexibility of polymers.[173] However, recent health concerns and increased industrial standards require more testing of the toxicity of these compounds. Thus, plasticizers such as dimethylsebacate (DMS), diethylsebacate (DES), and dibutylsebacate (DBS) were tested with DPPC,[173] which serves as a good model for the lung surfactant. The thermotropic data provided information on the extent of the interaction and potential penetration,[173] as large concentrations of plasticizers resulted in a complex transition and the coexistence of new phases and aggregates. Thus, these DSC-based results indicated negative health effects due to exposure to plasticizers.[173]

## Antimicrobial Peptides

The increasing presence of antibiotic-resistance bacterial strains has increased the interest in antimicrobial peptides.[174] In the field of novel peptide antibiotics, which are known to elicit their properties on the biological membranes, the study of peptide-lipid interactions is crucial in their design and development, as well as, in the understanding of molecular mechanisms.[174] Many peptides and analogs have been designed with the intention of specifically targeting bacterial phospholipid classes. As mentioned earlier, the peptide-lipid interaction can be observed based on a change in the phase transition. DSC has provided quantitative information on the effect of the peptide interaction on the membrane structure by comparing the thermotropic data of the lipid blank and the sample with the peptide in a concentration dependant manner.[174] Immediately evident from the thermogram’s preferential interaction with different lipid classes provides an insight into the type of binding.[174] Furthermore, the role of membrane perturbation and surface defects caused by protein-lipid interactions has implicated antimicrobial peptide activity based on phase separation, charge-charge interaction, membrane curvature strain, pore formation, and even detergent style effects.[174]

Different strains of bacteria have different phospholipid compositions in their membrane. However, the primary phospholipids are PG, PE, and CL. Membranes composed of PG have been used as the main model for binding to bacterial cell membranes.[125] Depending on the species and whether the bacteria are gram negative or positive, the percentage of PG can range from 6 – 90%, with just as large ranges for PE and CL.[174] Mammalian erythrocyte membranes contain mainly PC and SM on the outer leaflet and PE and PS are found in the inner leaflet.[174] Using different biomimetic lipid mixtures, liposomes can be used as a model of either human or bacterial membranes.

Prior DSC studies have shown that depending on the antimicrobial peptide there may be preferential interaction with certain lipid components.[174] This has been documented with the peptide cinnamycin specifically interacting with PE and sapecin interacting with cardiolipin.[174] However, the affinity of antimicrobial peptides has been predominantly to negatively charged phospholipids such as magainins, with greater bactericidal activity for membranes with higher PG concentrations.[174,175] Studies with different antimicrobial peptides have shown a preference for binding PG as opposed to other negatively charged lipids, such as, phosphatidic acid, phosphatidylserine, and cardiolipin.[176] Phase separation was consistently seen with the different peptides and PE / PG mixtures, with a preferential interaction with PG. Less pronounced thermotropic changes were predicted to be due to the rigidity of the CL membrane reducing peptide penetration.[176] DSC has also been effective in showing that the interaction is not purely electrostatic, as strong hydrocarbon chain disruption is evident.[176] Lipid specificity and effects of lipid chain length have been identified with protegrin-1, as only minor thermotropic changes with PA have been observed, whereas, major perturbations were observed with PG.[177] Interaction with peptide-rich and peptide-poor regions are observed in PG membranes in analogy to other antimicrobial peptides, such as HNP-2.[177] Furthermore, a decrease in enthalpy with added peptide concentration suggests a concentration-dependent binding.[177] Interaction is not solely headgroup-dependent as these effects seen with DMPG and DPPG are not mirrored with DSPG, as only a slight increase in T_m_ was recorded. This suggests an impact of lipid packing as the increased chain length results in more non-covalent interactions.[177]

Human neutrophils derived (Human Neutrophil Peptide) HNP-2 from the defensin class of peptides, showed a very high specificity for bacterial membranes over mammalian membranes.[178] This was accurately portrayed using model membranes and DSC with PC, SM, PE, and PG. Minimal changes to the PC or PC / SM mixtures were observed, with varying ratios of peptide, however, a proportional increase for the T_m_ of DPPG was found for the main transition.[178] Such behavior was consistent with a preferential stabilization of the gel phase by HNP-2. This was unique, as the majority of amphipathic peptides had limited interaction with the gel phase.[178] Gel phase interaction was confirmed by using DSC, by incubating HNP-2 / DPPG mixtures above and below the T_m_ and measuring the heat capacity of the L_α_ transition. Similar values were obtained with both incubations suggesting strong electrostatic interactions.[178] Such behavior has also been observed for tachyplesin I from horse shoe crab, which has a rigid β-sheet structure due to disulfide bridges.[178]

Differential scanning calorimetry was also used to show PG lipid segregation upon interaction with PGLa or HNP-2 from peptide-rich and peptide-poor domains, with a new transition occurring above the original T_m._[179] The peptide had a preferential interaction with PG liposomes, showing phase separation with peptide-rich and peptide-poor domains for PE / PG and PG membranes.[180] Furthermore, minimal peptide interaction was observed for biomimetic eukaryotic vesicles.[174] The peptides effect on the main transition was the aspect primarily studied using DSC, however, changes to the pretransition were also used to implicate packing defects caused by peptides.[181] Cathelicidins, indolicidin, and tritrpticin were all studied with varying model vesicle systems, such as, DMPC, DMPC / cholesterol, and DMPG, evaluating the different modes of interaction. In addition to abolishing the pre-transition of PC, cholesterol / PC demixing was observed suggesting the possibility of peptide-induced cholesterol domains.[182]

Opposite effects have been observed for protegrin-1, where new phase transition occurred below the T_m_. Such examples elucidate the ability of scanning calorimetry to establish the induction of lateral separation into domains as a possible mechanism of action.[179] This suggest a preferential interaction with PG components of bacterial membranes resulting in a thermogram with strong PE contribution.[179] Such behavior has been recorded for many antimicrobial peptides such as magainin II, buforin II, tachyplesin, protegrin I, and gramicidin S.[179] Such similarities between peptides that adopt different conformations, suggest that this may be a key trait to search for, when screening potential peptides using DSC. Furthermore, this indicates the crucial role of PG interaction and domain formation for bactericidal effects.[179]

Peptide LL-37, a member of the cathelicidin family, also showed chemotactic action on cancer and transformed cells.[183] With its high cationic charge (+ 6) and its alpha helical propensity upon binding to lipid membranes, its interaction with various model membranes was studied via DSC.[183,184] Typical loss of pre-transition followed by lower cooperativity prior to the formation of multi-phase transition[184] identified that the peptide interacted with the bilayer core of the membrane, reducing cooperativity and disrupting lipid packing.[184] The peptide was shown to be highly active reducing the sharp cooperative DPPC transition to a broad endotherm with a reduced enthalpy at an LL-37 ratio of 4%, correlating to small and wide angle X-ray scattering (SAXS). This broad region had been identified as the lamellar state, changing to disk-like micelles.[183] The peptide induced an even stronger effect with DPPG, with the main transition replaced with two overlapping transitions that were consistent with interdigitated and non-interdigitated domains.[183] The calorimetric data was utilized to make a phase diagram, indicating that the ability to form the quasi-interdigitated state depended on the helix topology, angle of hydrophobicity, and cationic distribution.[183] Interdigitation has also been observed with other peptides such as melittin, however, this was found with PCs at a mol ratio of 8%.[183]

Formations of other peaks is quite common, as observed for cecropin B and B3.[185] Such interactions have been found to be concentration-dependent, with low concentrations (~1 µM) typically broadening the profile, whereas, 20 times higher concentrations result in two shoulders above and below the pure lipid phase transition.[185] It has been suggested that the two phase transitions result from aggregation due to high peptide concentrations and pore formation, where two populations exist, one for lipids in the pore formation and the other for lipids not in the pore formation. Such examples of multiple peaks are found in defensins, magainins, and gramicidin S.[185]

Poly(L-lysine) or polyarginine peptides were used as model peptides to study the electrostatic interaction between peptides and different model lipid systems.[186] One such study used poly(L-lysine) peptides with various anionic lipids and lipid mixtures, which were studied via DSC.[186] Using model membranes the effects of chain length, peptide concentration, and charge density were investigated with DPPG or DPPG / DPPC or DPPG / DMPC membranes.[186] Peptide binding increased the T_m_ of the pure DPPG lipid with a stronger shift for longer peptides, probably from charge stabilization of the PG lipids in the gel phase. Mixed PC and PG systems yielded similar trends for other antimicrobial peptides inducing phase separation in the gel phase of the membrane preferentially interacting with the PG in the system leaving a relatively undisrupted PC L_α_ domain.[186]

**Figure 8:**
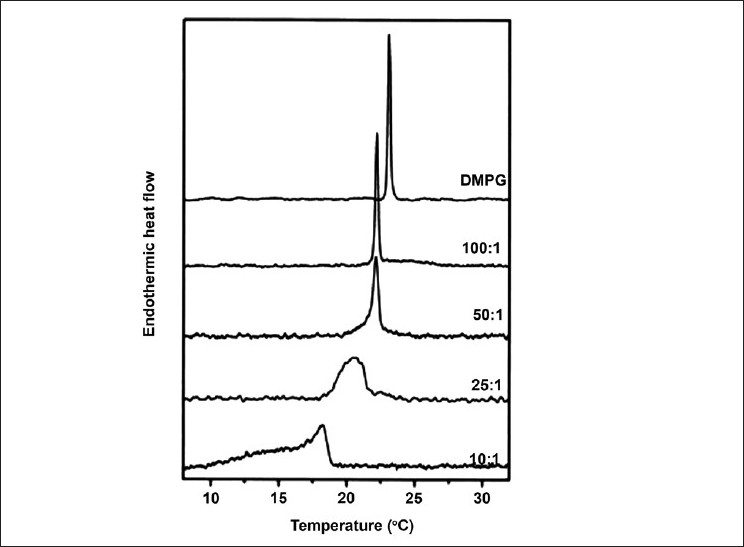
DSC endotherms for the given lipid to peptide ratios for Gramicidin S and DMPG MLVs. Reprinted from Biochimica et Biophysica Acta (BBA) – Biomembranes, Vol. 1417 Iss. 2, E.J. Prenner, R.N.A.H. Lewis, L.H. Kondejewski, R.S.Hodges, R.N. McElhaney, Differential scanning calorimetric study of the effect of the antimicrobial peptide gramicidin S on the thermotropic phase behavior of phosphatidylcholine, phosphatidylethanolamine and phosphatidylglycerol lipid bilayer membranes, 211-223, 1999, with permission from Elsevier[193]

Differential scanning calorimetry studies of the antimicrobial peptide Gramicidin S and MLVs composed of DMPC, DMPG, and DMPE have shown the dependence of interaction on the charge and structure of the headgroup[174] [Figure 8]. In respect to T_m_, enthalpy, and cooperativity, the peptide induced the largest differences with DMPG, whereas, DMPC had a moderate, and DMPE a comparably lower extent of interaction.[174] Interestingly, through repeated heating cycles of DSC, the GS bound to the DMPG vesicles protected the phospholipids from hydrolysis, due to their consistent exposure to high temperature[174] [Figure 8], suggesting a localization close to the phosphate groups. GS was also shown to suppress the pre-transition of DMPC at very low concentrations, increasing the width of the transition, which clearly indicated a perturbation of the bilayer.[187] Furthermore, GS induced an irreversible conversion to the L_α_ / HII phase with PE species after exposure to high temperatures, confirmed via ^31^P-NMR spectroscopy.[187] However, these results were not observed with the PG or PC species, indicating that the non-lamellar phase formation was headgroup-dependent.[187] Other peptides, such as protegrin-1 and PGLa also induced nonlamellar phases, like the cubic phase seen with GS.[183] For a recent review on nonlamellar phases and antimicrobial activity refer to.[188]

The number of scans required for the peptide-liposome interaction to equilibrate has been useful in determining the strength of the interaction as well as possible peptide redistribution.[182] This provides an insight into the mode of binding as DSC thermograms with tryptophan-rich cathelicidin show a progressive decrease in enthalpy and cooperativity of a specific component of the phase transition with each scan.[182] This can relate to the reduction of peptide-rich and peptide-poor regions, with increased cycling, due to increased lipid-peptide interaction. Also slow equilibration could be related to the possible release of peptide from the bilayer, upon cycling, indicating low affinity.[182]

Antimicrobial peptide lytic activity was studied with the help of DSC. Using the phase separation data with liposomes composed of PE and CL, it was observed that different antimicrobial peptides were efficient in separating PE from CL seen with two PE populations, one resembling pure PE and the other a CL-containing broadened PE peak.[98,105,131] Furthermore, a preferential interaction of peptides with certain membrane components was observed, based on the presence of peptide-free and peptide-bound domains.[98,105,131] Based on thermotropic data the effects of hydrocarbon backing, headgroup mismatch, membrane curvature, bilayer destabilization, and electrostatic repulsion had all been shown to be relevant for peptide vesicle interaction.[174] DSC has shown that antimicrobial peptides discriminate between different types of phospholipids, have a preferential interaction with bacterial PG membranes, and show little interaction with major components of mammalian membranes.

However, cases have been reported where minimal shouldering regions are observed, suggesting that there may be an equal distribution of peptides within the membrane, hence, all the lipids have a similar effect on the phase transition.[176] Furthermore, a complete translocation of the peptide may have occurred where the kinetics of peptide pore formation has been rapid, causing the peptide to reach an equilibrium on the outside and inside of the cell resulting in minimal changes to phase transition.[176] However, such possibilities can be determined by comparing the first and subsequent DSC scans.

Differential scanning calorimetry was also used to study peptide-induced membrane curvature. MSI-78, an analog from the naturally derived magainin class of antimicrobial peptides was added to different PE species to monitor the L_α_ / H_II_ phase transition.[189] Initially for 1,2-dipalmitoleoyl-phosphatidylethanolamine (DiPoPE) the T_H_ transition occurred at 43°C. Small additions of peptide increased the transition with 0.4% peptide to 46.4°C.[189] Such experiments were confirmed using ^31^P-NMR, showing that the peptide induced a positive curvature strain of DiPoPE membranes, while the peptide prevented POPE from transforming from the lamellar state even at high concentrations.[189] Low peptide concentrations of MSI-78 resulted in morphology changes of the bilayer, consistent with pore formations, whereas, high concentrations typically increased the population of the lipids in the hexagonal phase.[189]

Insight into peptide penetration can also be determined based on the degree of disruption to the lipid acyl chains. Furthermore, comparison to other antimicrobial systems has been used to establish the mechanism. Such evaluations have been made for magainin, showing less disruption to acyl chains of DPPG compared to melittin, suggesting less penetration into the hydrocarbon region.[190]

## Summary

Differential Scanning Calorimetry remains one of the primary tools for thermodynamic analysis. The rapid progression to modern nano and automated DSC instruments has shown the development and improvements in multiple applications. Advancements with software allow for easy interpretation of thermodynamic data, which again make DSC a very attractive technique. The instrument is comparatively inexpensive and the high sensitivity models only require a relatively dilute suspension in the aqueous phase.

Even as most studies focus on protein conformation, DNA binding, lipid studies, and lipid-peptide or lipid-protein interaction, DSC has also been used in industry testing, health concern with plasticizers, and biomimetic lung membranes.[173] DSC is one of the most powerful techniques for the routine measurement of gel-to-liquid crystalline phase transition in the lipid bilayers and biological membranes, and changes in interactions of antimicrobial peptides are used to assess peptide-membrane interactions.[174] Applications to the pharmaceutical industry such as drug purity, stability, DNA drugs, lipid targets and different drug delivery models have improved our ability to study different compounds.[32] The ability to determine the physical and energetic properties of a compound has made DSC increasingly popular in drug development.[32]
